# Magnesium and the Risk of Cardiovascular Events: A Meta-Analysis of Prospective Cohort Studies

**DOI:** 10.1371/journal.pone.0057720

**Published:** 2013-03-08

**Authors:** Xinhua Qu, Fangchun Jin, Yongqiang Hao, Huiwu Li, Tingting Tang, Hao Wang, Weili Yan, Kerong Dai

**Affiliations:** 1 Shanghai Key Laboratory of Orthopaedic Implant, Shanghai Ninth People’s Hospital, Shanghai Jiaotong University School of Medicine, Shanghai, China; 2 Department of Pharmacology and Biostatistics, Institute of Medical Sciences, Shanghai Jiaotong University School of Medicine, Shanghai, China; 3 Department of Health Policy and Management, Johns Hopkins Bloomberg School of Public Health, Baltimore, Maryland, United States of America; Universidad Peruana Cayetano Heredia, Peru

## Abstract

**Background:**

Prospective studies that have examined the association between dietary magnesium intake and serum magnesium concentrations and the risk of cardiovascular disease (CVD) events have reported conflicting findings. We undertook a meta-analysis to evaluate the association between dietary magnesium intake and serum magnesium concentrations and the risk of total CVD events.

**Methodology/Principal Findings:**

We performed systematic searches on MEDLINE, EMBASE, and OVID up to February 1, 2012 without limits. Categorical, linear, and nonlinear, dose-response, heterogeneity, publication bias, subgroup, and meta-regression analysis were performed. The analysis included 532,979 participants from 19 studies (11 studies on dietary magnesium intake, 6 studies on serum magnesium concentrations, and 2 studies on both) with 19,926 CVD events. The pooled relative risks of total CVD events for the highest vs. lowest category of dietary magnesium intake and serum magnesium concentrations were 0.85 (95% confidence interval 0.78 to 0.92) and 0.77 (0.66 to 0.87), respectively. In linear dose-response analysis, only serum magnesium concentrations ranging from 1.44 to 1.8 mEq/L were significantly associated with total CVD events risk (0.91, 0.85 to 0.97) per 0.1 mEq/L (P_nonlinearity_ = 0.465). However, significant inverse associations emerged in nonlinear models for dietary magnesium intake (P_nonlinearity_ = 0.024). The greatest risk reduction occurred when intake increased from 150 to 400 mg/d. There was no evidence of publication bias.

**Conclusions/Significance:**

There is a statistically significant nonlinear inverse association between dietary magnesium intake and total CVD events risk. Serum magnesium concentrations are linearly and inversely associated with the risk of total CVD events.

## Introduction

Cardiovascular disease (CVD) is a major cause of death and disability worldwide [1], [2]. The prevalence of CVD is increasing rapidly, and the need for prevention is widely acknowledged [1], [3]–[5]. Increased physical activity, tobacco control, and weight control are key steps in the prevention of CVD, but insights into the role of other lifestyle factors, may contribute to additional prevention strategies [6]–[11].

Magnesium is the fourth most abundant mineral found in the body and is considered to be favorably associated with the risk of CVD [12]. However, low consumption of magnesium is common throughout the world. In the United States, the prevalence of inadequate magnesium intake for adults is about 64% among males and 67% among females; among individuals aged more than 71 years, the figure rises to 81% and 82% for males and females, respectively [13].

Current guidelines for the prevention of CVD from the American Medical Association (AMA) include goals for magnesium intake [14]. These guidelines suggest that magnesium-rich foods have a positive effect on blood pressure (BP) [14]. Recently, several literature reviews and editorials have focused on the relevance of magnesium in CVD. These reviews indicate that the prevalence of CVD events caused by inadequate magnesium intake and low serum magnesium concentrations has been underestimated and that cardiovascular health could be related to magnesium intake [15]–[17].

Although higher dietary magnesium intake and serum magnesium concentrations are plausibly linked to a reduced risk of CVD events, the absence of randomized clinical trials on this topic and inconsistency among the findings of prospective cohort studies [18]–[36] preclude definitive recommendations at present. Meta-analysis is an important tool for revealing trends that might not be apparent and should always be used to assess the association between factors and total CVD events risk [2], [37]–[41]. Moreover, meta-analyses that focus on the risk of total CVD events are useful for the establishment of clinical policies and guidelines.

Therefore, we conducted a meta-analysis of prospective studies for the following purposes: (1) to examine the categorical association between dietary magnesium intake and serum magnesium concentrations and the risk of total CVD events; (2) to quantify a dose-response pattern of dietary magnesium intake and serum magnesium concentrations on total CVD risk; and (3) to examine the shape of the dose–response relationship by conducting linear and nonlinear dose-response analyses.

## Methods

We performed a systematic review of the existing literature, followed by a meta-analysis of prospective cohort studies according to the MOOSE guidelines [42] and the PRISMA statement [43], [44].

### Data Sources and Searches

We performed a systematic literature search of MEDLINE, EMBASE, and OVID up to February 1, 2012, without limits. All searches were performed using medical subject headings (MeSH) or free text words. We combined search terms for the outcomes (cardiovascular disease, stroke, cerebral infarction, intracerebral hemorrhage, subarachnoid hemorrhage, cerebrovascular accident, myocardial infarction, heart attack, ischemic heart disease, coronary artery disease, mortality, death, fatality, and fatal), effect measures (magnesium intake, magnesium supplement, dietary magnesium, total magnesium, blood magnesium, and serum magnesium), and risk estimates (odds ratio, relative odds, risk ratio, relative risk, and hazard ratio). In addition, we hand-searched the reference lists of primary studies, review articles, and clinical guidelines. We inspected the full text of any citation that appeared relevant. Moreover, we also hand-searched abstract of meetings related to Nutriology and Cardiology which provided printed or electronic publications. However, none of these meeting abstracts was quoted in this study.

### Study Selection

Two reviewers independently evaluated studies for inclusion. Discrepancies between their decisions regarding study inclusion and interpretation of data were resolved by arbitration, and consensus was reached after discussion. Studies were included in the meta-analysis if they met the following criteria: (1) prospective design; (2) adult population (age, >18 years); (3) the exposure of interest was intake of magnesium or serum magnesium concentrations; (4) the outcome of interest was CVD events, and (5) the risk estimates, such as relative risks, odds ratios, or hazard ratios that could be transformed into relative risks with 95% confidence interval were reported. Studies that did not meet the inclusion criteria were excluded during the initial review phase.

### Data Extraction

All data were independently abstracted by 2 reviewers using a standardized data collection form. Discrepancies were resolved through discussion with other investigators and through reference to the original articles. The following data were extracted from each study: the first author’s last name, publication year, country where the study was performed, year of follow-up, recruitment time, participant sex and age, sample size, number of cases, reported outcome, method of outcome assessment, measure and range of exposure, variables adjusted for in the analysis, and risk estimates with corresponding confidence intervals for each category of dietary magnesium intake and serum magnesium concentrations and/or as a continuous variable. We extracted the relative risks and 95% confidence intervals that reflected the greatest degree of control for potential confounders for use in the main analyses. Our main outcome was the association with total CVD events, comprising stroke, coronary heart disease (CHD), and CVD death [37], [38], [40].

### Data Synthesis and Analysis

We used relative risks as the common measure of association across studies. Hazard ratios and odds ratios were transformed into relative risks [45]–[47]. To summarize the association of magnesium with the risk of total CVD events, the effect measures were pooled for the highest vs. lowest categories for dietary magnesium intake or serum magnesium concentrations. We also evaluated the dose-response relationship between dietary magnesium intake, serum magnesium concentrations, and total CVD events. For the included studies where categories were used, we estimated a relative risk as a continuous variable for a 100 mg/d increase in dietary magnesium intake and a 0.1 mEq/L increase in serum magnesium concentrations based on the method described by Greenland and Longnecker, which takes into account level-specific relative risks [48], [49]. For articles that did not provide median or mean intakes per category, we assigned the midpoint of the upper and lower boundaries of each category as the average intake. When the lowest or highest category was open-ended, we assumed the open-ended interval length to be the same as the closest category. We used restricted cubic splines (3 knots at fixed percentiles of 10%, 50%, and 90% of the distribution) to examine potential nonlinear dose-response associations between dietary magnesium intake, serum magnesium concentrations, and the risk of total CVD events [50], [51]. A probability value for nonlinearity was calculated by testing the null hypothesis that the coefficient of the second spline is equal to 0 [51].

For the meta-analysis, both a fixed-effects model (weighted with inverse variance) and a random-effects model were considered [52]. Heterogeneity between studies was assessed using Cochran Q statistics and I^2^ statistics [53]. As suggested by Higgins et al, I^2^ values of 25%, 50%, and 75% were considered low, moderate, and high, respectively [54]. For P<0.10 values of the Cochran Q statistic, it was considered statistical heterogeneity, and a random-effects model was reported. Subgroup and meta-regression analyses were used to identify associations between the risk of total CVD events and relevant study characteristics (individual CVD outcomes, sex of participants, country of origin, distribution fractions of magnesium intake or serum magnesium concentrations, magnesium difference, period of follow-up, number of participants, number of CVD events, and incidence of CVD events) as possible sources of heterogeneity. Subgroup analysis was used for classified variables, and meta-regression analysis was used for continuous variables. Funnel plot asymmetry was used to detect publication bias, and the Egger regression test was used to measure funnel plot asymmetry [55]. We also performed the “trim and fill” procedure to further assess the possible effect of publication bias in our meta-analysis. This method considers the possibility of hypothetical “missing” studies that might exist, imputes their relative risks, and recalculates a pooled relative risk that incorporates the hypothetical missing studies as though they actually existed [56], [57].

All analyses were conducted using Stata 10 (StataCorp, College Station, Texas).

## Results

The detailed steps of our literature search are shown in supporting information; see Figure S1. We performed a systematic literature search of MEDLINE, EMBASE, and OVID. There were 2478 articles identified from database search. After evaluations of titles and abstracts, we excluded 2443 studies, 480 of which were duplicates, while another 1963 did not satisfy criteria. Then we retrieved the remaining 35 articles for eligibility, and excluded 16 of them because of cross-sectional (n = 13) and using odds ratio without CIs (n = 3). After the screening and deletion, 19 articles was left and used in this meta-analysis finally. [18]–[34]. Agreement between observers regarding inclusion of studies was considered to be good (Cohen’s unweighted κ = 0.93).

### Description of Study Characteristics

The characteristics of the included prospective cohort studies are summarized in Tables 1 and 2. There were 19 prospective cohort studies with 532,979 participants and over 19,926 CVD events, including 6668 strokes, 5836 cases of CHD outcomes, and 5339 CVD deaths. The cohorts were from 7 different countries (12 studies from the United States [18]–[23], [25], [27], [29], [30], [32], [33], 2 from Sweden [26], [28], and 1 each from Finland, France, Germany, Japan, and China [24], [31], [34]–[36]). Nine studies recruited both male and female participants [19], [25], [29], [30], [32]–[36], whereas 6 recruited only males [18], [21], [22], [24], [26], [31] and 4 recruited only females [20], [23], [27], [28]. The age of participants was ≥25 years. Study lengths ranged from 7 to 30 years. Eleven of 19 cohort studies only reported the dietary magnesium intake [18], [20]–[24], [26]–[28], [35], [36], 6 only reported the serum magnesium concentration [29]–[34], and 2 reported both [19], [25].

**Table 1:** Characteristics of the prospective studies included in the meta-analysis of published studies on magnesium intake and the risk of CVD events.

Source	Location/Follow-up, y	Study Population (n)	Sex/Age, y	Recruitment Time	Outcome(s)	Outcomes Assessment	Magnesium intake Assessment
Ascherio (1998)	United States, 8	43,738	Male, 40–75	1986	Stroke	Ascertained by self-report; subclassified accordingto the criteria of the National Survey of Stroke.	Validated FFQ
Liao (1998)	United States, 7	13,922	Female/Male, 45–64	1987–1989	CHD	Ascertained by self-report and medical records, deathcertificates; reviewed by members of the ARIC Morbidityand Mortality Classification Committee	FFQ
Iso (1999)	United States, 14	85,764	Female, 34–59	1976	Stroke	Ascertained by self-report and medical records;confirmed according to the criteria of theNational Survey of Stroke.	Validated FFQ
Abbott (2003)	United States, 30	7172	Male, 45–68	1965–1968	CHD	Confirmation by the Honolulu Heart ProgramMorbidity and Mortality Review Committee.	24-hour dietary recall
Al-Delaimy (2004)	United States, 12	39,633	Male, 40–75	1986	CHD	Ascertained by self-report and medical records.	Validated FFQ
Song (2005)	United States, 10	39,876	Female, 39–89	1991	CVD	Confirmed through medical records, autopsy reports,and death certificates.	Validated FFQ
Larsson (2008)	Finnish,13.6	26,556	Male, 50–69	1985–1988	Stroke	Identified by National Hospital Discharge Register andthe National Register of Causes of Death and classifiedaccording to ICD-8,9,10 (ICD-8 codes 430–434,436; ICD-9codes 430, 431, 433, 434, 436; ICD-10 codes I60, I61, I63,and I64)	Validated FFQ
Weng (2008)	China, 10.6	1772	Female/Male, ≥40	1990–1993	Stroke	Ascertained by self-reported; classified according toICD-9-CM (codes 430 to 438)	FFQ
Ohira (2009)	United States, 15	14,221	Female/Male, 45–64	1987–1989	Stroke	Ascertained by self-report, local hospitals, state vitalstatistics offices; and classified according to ICD-9(codes 430–438)	FFQ
Kaluza (2010)	Swedish, 10	23,366	Male, 45–79	1997–1998	CVD death	Review of Swedish Death and Population Registersand classified according to ICD-10 (codes I00–I79)	Validated FFQ
Chiuve (2011)	United States, 10	88,375	Female, 30–55	1976	CVD death	Documented by medical records	FFQ
Larsson (2011)	Swedish,10.4	34,670	Female, 49–83	1987–1990	Stroke	Ascertained by Swedish Hospital Discharge Registry andidentified according to ICD-10 (codes I60, I61, I63, I64)	Validated FFQ
Zhang (2012)	Japan, 14.7	58,615	Female/Male, 40–79	1988–1990	CVD death	A systematic review of death certificates; and classifiedaccording to ICD-9 (codes 390–459) and ICD-10 (codes101–199)	Validated FFQ

CHD = coronary heart disease; CVD = cardiovascular disease; FFQ = food frequency questionnaire; ICD = International Classification of Diseases.

**Table 2:** Characteristics of prospective studies included in meta-analysis of published studies on serum magnesium concentrations and total CVD risk.

Source	Location/Follow-up, y	Study Population(n)	Sex/Age, y	RecruitmentTime	Outcome(s)	Outcomes Assessment	Serum magnesium Assessment
Gartside (1995)	United States, 10	8251	Female/Male, 25–74	1971–1975	CVD	Adjudicated by hospital records and death certificates; classified according to ICD-9 (codes 390–459).	NA
Liao (1998)	United States, 7	13,922	Female/Male, 45–64	1987–1989	CHD	Ascertained by self-report and medical records, death certificates; reviewed by members of the ARIC Morbidityand Mortality Classification Committee	Measured the procedure of Gindler and Heth,and used the metallochromic dye calmagite.
Ford (1999)	United States, 19	12,340	Female/Male, ≥25	1971–1975	CHD, CVDdeath	Determined by the health care facility records and death certificate; classified according to ICD-9 (codes 410–414).	Measured by atomic absorptionspectrophotometry using the method ofHansen and Freier.
Leone (2006)	France, 18	4035	Male 30–60	1980–1985	CVD death	Determined by the death certificate classified according to ICD-9 (codes 390–459) and ICD-10 (codes I00–I99).	Measured by flame atomic absorptionspectrometry.
Ohira (2009)	United States, 15	14,221	Female/Male, 45–64	1987–1989	Stroke	Ascertained by self-report, local hospitals, state vital statistics offices; classified according to ICD-9(codes 430–438).	Based on the procedure of Gindler and Hethand used the metallochromic dye, calmagite.
Khan (2010)	United States, 20	3531	Female/Male, 44.3±10	1971	CVD, CVDdeath	Adjudicated by hospital records, medical office notes, and Framingham clinic visit notes.	Measured using a standard colorimetric assay.
Peacock (2010)	United States, 12	14,232	Female/Male, 45–64	1987–1989	CVD death	Ascertained by self-report, hospitals, and death certificates.	Performed at visits 1 and 2 and was based onthe procedure of Gindler and Heth usingthe metallochromic dye calmagite.
Reffelmann (2011)	Germany, 10.1	3910	Female/Male, 20–79	1997–2001	CVD death	Assessed by Sociodemographic and medical histories; defined with ICD-10 codes I10–I79.	Performed on the Siemens HealthcareDiagnostics using a modification of MTB complexiometricprocedure.

MTB = methylthymol blue; NA = not available.

Most studies used food-frequency questionnaires (FFQs) for dietary assessment. For dietary magnesium intake, the mean intakes of magnesium for highest categories were 468 mg/d and for lowest categories were 223 mg/d. For serum magnesium, the mean concentrations of serum magnesium for highest categories were 2.07 mEq/L and for lowest categories was 1.36 mEq/L. The most frequent confounders that were adjusted for aside from age included body mass index (BMI), physical activity, smoking status, alcohol consumption, history of diabetes, dyslipidemia, and BP status or history of hypertension. Adjustments for potential dietary confounders varied across individual studies. Fiber intake was adjusted for in 4 studies [18], [22], [26], [28], and potassium intake was adjusted for in 3 studies [18], [22], [27].

### Association between Dietary Magnesium Intakes and Total CVD Events Risk

**Figure 1:**
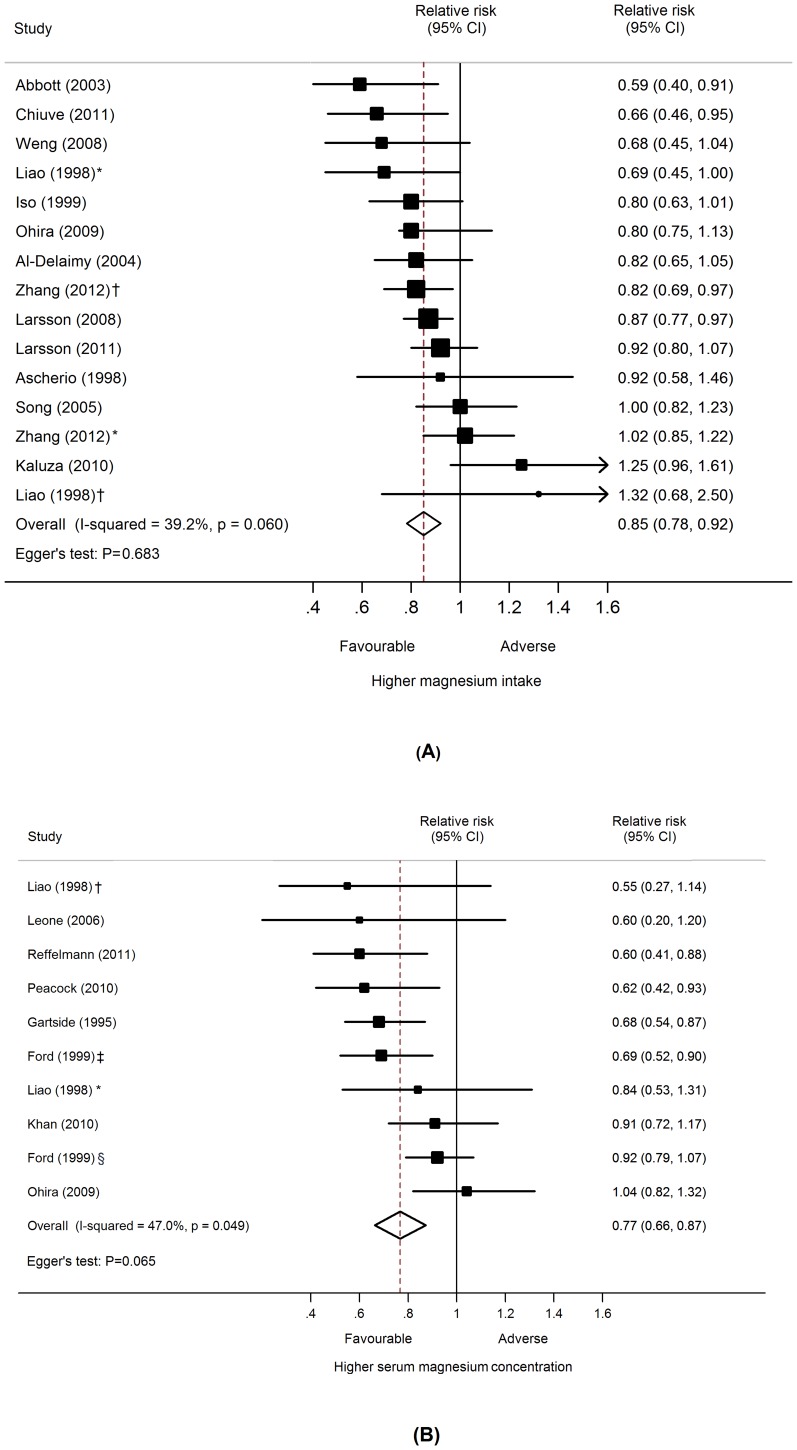
Dietary magnesium intake, serum magnesium concentrations, and the risk of total CVD events. (A) Dietary magnesium intake; (B) Serum magnesium concentrations. Adjusted relative risks for the association between dietary magnesium intake and serum magnesium concentrations (highest vs. lowest categories) and the risk of total CVD events were sorted by statistical size, defined by the inverse of the variance of the relative risks. CI = confidence interval; RR = relative risk. *Male participants. †Female participants. ‡CVD death outcomes. §CHD outcomes.

Thirteen prospective cohort studies [18]–[28], [35], [36] with 477,680 participants and over 14,918 CVD events were included in this analysis (Table 3). The multivariable-adjusted relative risks for each study and all studies combined for the highest vs. lowest categories of dietary magnesium intake are shown in Figure 1. In the meta-analysis, there was a statistically significant inverse relationship between higher dietary magnesium intake and risk of total CVD events such that risk of CVD was 15% lower among individuals with the highest intake of magnesium than among those with the lowest intake (relative risk 0.85, 95% confidence interval 0.78 to 0.92; P<0.001). There was moderate heterogeneity across studies (P* = *0.060; I^2^ = 39.2%). Sensitivity analysis showed that the pooled estimate of the effect of dietary magnesium intake on risk of total CVD events did not vary substantially with the exclusion of any one study.

**Table 3:** Detailed outcomes of each the published studies included in the meta-analysis.

Source	Persons	Cohort (n)	Classification of CVD	No. of CVD	RR (95% CI) of CVD	Comparison	The highest vs. lowest categories*	Factors Controlled for in Multivariate Analysis
**Dietary Magnesium Intake**								
Ascherio(1998)	Male	43,738	Stroke	328	0.92 (0.58, 1.46)	Quintile(V vs. I)	452 vs. 243 (median)	Age, total intake of energy, potassium and fiber, smoking, alcohol consumption, history of hypertension, hypercholesterolemia, parental history of myocardial infarction, profession, BMI and PA.
Liao(1998)	Male Female	6155 7767	CHD	223 96	0.69 (0.45, 1.0)1.32 (0.68, 2.5)	Quartile(IV vs. I)	Range: 39.9–485.1	Age, race, ARIC field center, smoking status, cigarette-years, alcohol drinking, PA, education level, fibrinogen, total cholesterol, HDL-C, triglycerides, WHR, diuretic use status, hormone replacement, SBP and diabetes status
Iso(1999)	Female	85,764	Stroke	690	0.80 (0.63, 1.01)	Quintile(V vs. I)	381 vs. 211 (median)	Age and smoking status.
Abbott(2003)	Male	7172	CHD	1431	0.59(0.40,0.91)	Quintile(V vs. I)	340–1138 vs. 50.3–186	Age, BMI, the confounding dietary variable(s), total cholesterol, hypertension, diabetes, PA, smoking status, and alcohol intake.
Al-Delaimy (2004)	Male	39,633	CHD	1449	0.82 (0.65, 1.05)	Quintile(V vs. I)	453 vs. 261 (median)	Age, BMI, time period, energy intake, history of diabetes, history of high cholesterol, smoking status, aspirin intake, family history of MI, vitamin E, alcohol, PA, and nutrient variables (trans fatty acid, total protein, cereal fiber, folate, omega 3 fatty acid, potassium).
Song(2005)	Female	39,876	Total CVD events	1037	1.00 (0.82–1.23)	Quintile(V vs. I)	433 vs. 255 (median)	Age, BMI, randomized treatment assignment, total energy, smoking, PA, alcohol, multivitamin use, postmenopausal hormone use, history of diabetes, hypertension, hypercholesterolemia, and MI.
Larsson(2008)	Male	26,556	Stroke	3281	0.87(0.77,0.97) †	Quintile(V vs. I)	575 vs. 382 (median)	Age, BMI, supplementation group, smoking, BP, serum total cholesterol, serum HDL-C, histories of diabetes and CHD,PA, alcohol, and total energy.
Weng(2008)	Female/Male	1772	Stroke	132	0.68 (0.45, 1.04)	Quartile(IV vs. I)	>282.2 vs. <242.6	Age, sex, smoking, area, central obesity, BMI, diabetes mellitus, physical activity, hypertension, use of antihypertensive drug, self-report heart disease, apolipoprotein B, hypercholesterolemia, hypertriglyceridemia, fibrinogen, plasminogen and alcohol intake
Ohira(2009)	Male/Female	14,221	Stroke	557	0.80 (0.75, 1.13)	Quartile(IV vs. I)	>367 vs. <186	Age, BMI, sex, race-field center, smoking, LDL-C, HDL-C, fibrinogen, vWf, education. SBP, antihypertensive medication, and diabetes.
Kaluza(2010)	Male	23,366	CVD death	819	1.25 (0.96, 1.61)	Thirds(III v I)	≧481 vs. <426	Age, marital, education, self-reported health status, smoking status, PA, WHR, dietary fiber, saturated fatty acid, phosphorus, alcohol, vitamin D, calcium and intake.
Chiuve(2011)	Female	88,375	CVD death	505	0.66 (0.46, 0.95)	Quartile(IV vs. I)	383 vs. 235 (median)	Age, BMI, history of CVD, total calories, smoking, parental history of myocardial infarction, alcohol, PA, use of aspirin, postmenopausal hormones, diuretics, calcium, potassium, and vitamin D, hypertension, diabetes, and hypercholesterolemia.
Larsson(2011)	Female	34,670	Stroke	1680	0.92 (0.80, 1.07)	Quintile(V vs. I)	373 vs. 267 (median)	Age, smoking status, pack-years of smoking, educational, BMI, PA, history of diabetes and hypertension, aspirin use, family history of MI, and intakes of total energy, alcohol, protein, cholesterol, total fiber, and folate.
Zhang(2012)	Male Female	23,083 35,532	CVD death	1343 1347	1.02 (0.85, 1.22)0.82 (0.69, 0.97)	Quintile(V vs. I)	294 vs. 173 (median)274 vs. 175 (median)	BMI, smoking status, ethanol intake, history of hypertension, history of diabetes, sports time, walking time, educational status and perceived mental stress, and for women, menopausal status and hormone replacement therapy.
**Serum Magnesium Concentrations**								
Gartside (1995)	Female/Male	8251	Total CVD events	492	0.68 (0.54,0.87)	Thirds(III v I)	≧1.74 vs. <1.62	Age, sex, quetelet index, PA, exercise, sedimentation rate, dietary iron, smoking, maximum weight, alcohol, and riboflavin.
Liao(1998)	Male Female	6155 7767	CHD	223 96	0.84 (0.53,1.31) 0.55 (0.27, 1.14)	Quartile(IV vs. I)	≧1.8 vs. ≦1.5	Age, race, ARIC field center, smoking status, cigarette-years, alcohol drinking, PA, education, fibrinogen, total cholesterol, HDL-C, triglycerides, WHR, diuretic use status, hormone replacement, SBP and diabetes status.
Ford(1999)	Female/Male	12,340	CHD CVD death	2637 1005	0.92 (0.79, 1.07) 0.69 (0.52, 0.90)	Quartile(IV vs. I)	≧1.77 vs. <1.59†	Age, sex, race, education, smoking status, cholesterol, SBP, diabetes, antihypertensive medication, BMI, PA, and alcohol consumption.
Leone(2006)	Male	4035	CVD death	56	0.6 (0.2–1.2)	Thirds(III v I)	Range: 0.477–3.538†	Age, BMI, smoking status, alcohol consumption, PA, LDL, HDL, cholesterol, triglycerides, diabetes, and CVD history.
Ohira(2009)	Male/Female	14,221	Stroke	557	1.04 (0.82, 1.32)	Quartile(IV vs. I)	≧1.8 vs. ≦1.5	Age, BMI, sex, race-field center, smoking, LDL-C, HDL-C, fibrinogen, vWf, education. SBP, antihypertensive medication, and diabetes.
Khan (2010)	Male/Female	3531	Total CVD events	554	0.91 (0.72, 1.17)	Quartile(IV vs. I)	NA	Age, sex, BMI, diabetes, SBP, smoking status, hypertension treatment, glomerular filtration rate hemoglobin, serum albumin, and total/HDL ratio.
Peacock(2010)	Male/Female	14,232	CVD death	264	0.62 (0.42–0.93)	Quartile(IV vs. I)	≧1.75 vs. ≦1.5	Age, race, sex, field center, HDL, LDL, TG, serum K, heart rate- adjusted QT interval, PA, smoking, pack-years, ETOH intake, education, diabetes, hypertension, and diuretics use.
Reffelmann (2011)	Male/Female	3910	CVD death	NA	0.60(0.41,0.88) †	Intergroup difference	NA	Age, sex, diabetes, smoking status, BMI, glomerular filtration rate, arterial hypertension, use of calcium antagonists, beta blocker, diuretics, statins, and ACE and angiotensin-receptor inhibitors.

BMI = body mass index; HDL-C = high density lipoprotein cholesterol; LDL-C = low density lipoprotein cholesterol; MI = myocardial infarction; PA = physical activity; SBP = systolic blood pressure; WHR = waist/hip ratio.

* Mg/d for dietary magnesium intake and mEq/L for serum magnesium concentrations.

† Recalculate from primal studies.

### Association between Serum Magnesium Concentration and Total CVD Events Risk

Eight prospective cohort studies [19], [25], [29]–[34] with information on 74,422 participants and over 5884 CVD events were included in this analysis (Table 3). Individuals in the highest category of serum magnesium concentration had an approximately 20% lower risk of total CVD events compared to those in the lowest concentration category (0.77, 0.66 to 0.87; P<0.001) with moderate heterogeneity between studies (P* = *0.049; I^2^ = 47%) (Figure 1). Sensitivity analysis showed that the pooled estimate of the effects of serum magnesium concentrations on risk of total CVD events did not vary substantially with the exclusion of any one study.

### Sources of Heterogeneity

#### Subgroup analysis

Within the subgroup analysis, we examined location as a possible source of heterogeneity. For dietary magnesium intake, the relative risks were 0.80 (0.72 to 0.88) for studies conducted in the United States, 0.91 (0.83 to 0.99) for studies in Europe, and 0.87 (0.76 to 0.97) for studies in Asia; significant interactions were observed between subgroups (P = 0.050). For serum magnesium concentrations, this analysis did not show any significant interaction between location variables (P = 0.075). For individual CVD outcomes, the relative risks were 0.73 for CHD (0.60 to 0.87), 0.87 for stroke (0.81 to 0.93), and 0.89 for CVD death (0.79 to 0.99) with no interaction between them (P = 0.060). For serum magnesium concentrations, the relative risks were 0.64 (0.52 to 0.77) for CVD death and 0.82 (0.73 to 0.92) for CVD and CHD, with a significant interaction between the subgroups (P = 0.021). We also examined sex as a possible source of heterogeneity for dietary magnesium intake. The relative risks were 0.87 (0.74 to 1.00) for males and 0.86 (0.76 to 0.95) for females, with a significant interaction between the subgroups (P = 0.048). For serum magnesium concentrations, this analysis did not show any significant interaction between sex variables (P = 0.797). We also examined distribution fractions of magnesium assessment as possible sources of heterogeneity. The results showed that there were no significant interactions between variables (Table 4).

**Table 4:** Subgroup analysis to investigate differences between studies included in meta-analysis.

Subgroup	Cohorts (n)	RR (95% CI)	Q	I^2^(%)	P value	P value for heterogeneity between subgroups
**Dietary Magnesium Intake**						
**Individual CVD outcomes**						
Stroke	7	0.87 (0.81, 0.93)	4.76	0	0.575	
CHD	3	0.73 (0.60, 0.87)	3.63	17	0.305	0.060
CVD death	3	0.89 (0.79, 0.99)	10.95	73	0.012	
**Sex**						
Male	7	0.87 (0.74, 1.00)	14.34	58	0.026	0.048
Female	6	0.86 (0.76, 0.95)	6.79	26	0.237	
**Location**						
United States	8	0.80 (0.72, 0.88)	9.70	18	0.287	
Europe	3	0.91 (0.83, 0.99)	4.84	59	0.089	0.050
Asian	2	0.87 (0.76, 0.97)	4.60	57	0.100	
**Distribution fractions**						
Quintile	8	0.87 (0.82, 0.93)	10.53	24	0.230	0.187
Quartile	4	0.74 (0.80, 0.90)	2.63	0	0.621	
**Serum Magnesium concentrations**						
**Individual CVD outcomes**						
CVD & CHD	4	0.82 (0.73, 0.92)	6.82	41	0.145	0.021
CVD death	4	0.64 (0.52, 0.77)	0.42	0	0.935	
**Sex**						
Male & Female	6	0.79 (0.72, 0.88)	15.24	61	0.018	0.797
Only male	2	0.75 (0.44, 1.06)	0.55	0	0.458	
**Location**						
United States	6	0.80 (0.73, 0.88)	13.81	49	0.055	0.075
Europe	2	0.78 (0.71, 0.81)	0.00	0	1.000	
**Distribution fractions**						
Quartile	5	0.84 (0.75, 0.92)	0.09	46	0.087	0.069
Thirds	2	0.67 (0.52, 0.83)	11.04	0	0.766	

#### Meta-regression analysis

Meta-regression analysis indicated no influence of difference in magnesium assessment, length of follow-up, number of participants or CVD events, or the incidence of CVD on the inverse association between dietary magnesium intake, serum magnesium concentrations, and the risk of total CVD events (Table 5).

**Table 5:** Meta-regression analysis.

	Dietary Magnesium Intake				Serum Magnesium Concentrations			
	Coefficient	SE	P Value	95% CI	Coefficient	SE	P Value	95% CI
**Magnesium difference**	0.001	0.000	0.797	−0.001 to 0.002	−1.821	0.993	0.318	−14.441 to 10.800
**Length of follow-up**	−0.008	0.013	0.585	−0.037 to 0.022	−0.164	0.111	0.377	−1.571 to 1.242
**No. of participants**	−0.002	0.002	0.411	−0.005 to 0.003	−0.445	0.243	0.319	−3.538 to 2.648
**No. of CVD events**	0.106	0.104	0.335	−0.130 to 0.342	0.396	0.219	0.322	−2.385 to 3.176
**Incidence of CVD**	−0.021	0.025	0.431	−0.078 to 0.036	−0.470	0.272	0.334	−3.923 to 2.983

### Publication Bias

There was no evidence of publication bias with regard to dietary magnesium intake or serum magnesium concentrations in relation to the risk of total CVD events for highest vs. lowest analysis, as indicated by the Egger test (dietary magnesium intake: P* = *0.64; serum magnesium concentrations: P* = *0.41) and the “trim and fill” method (Figure S2).

### Dose-response Analysis

#### Dietary magnesium intake

**Figure 2:**
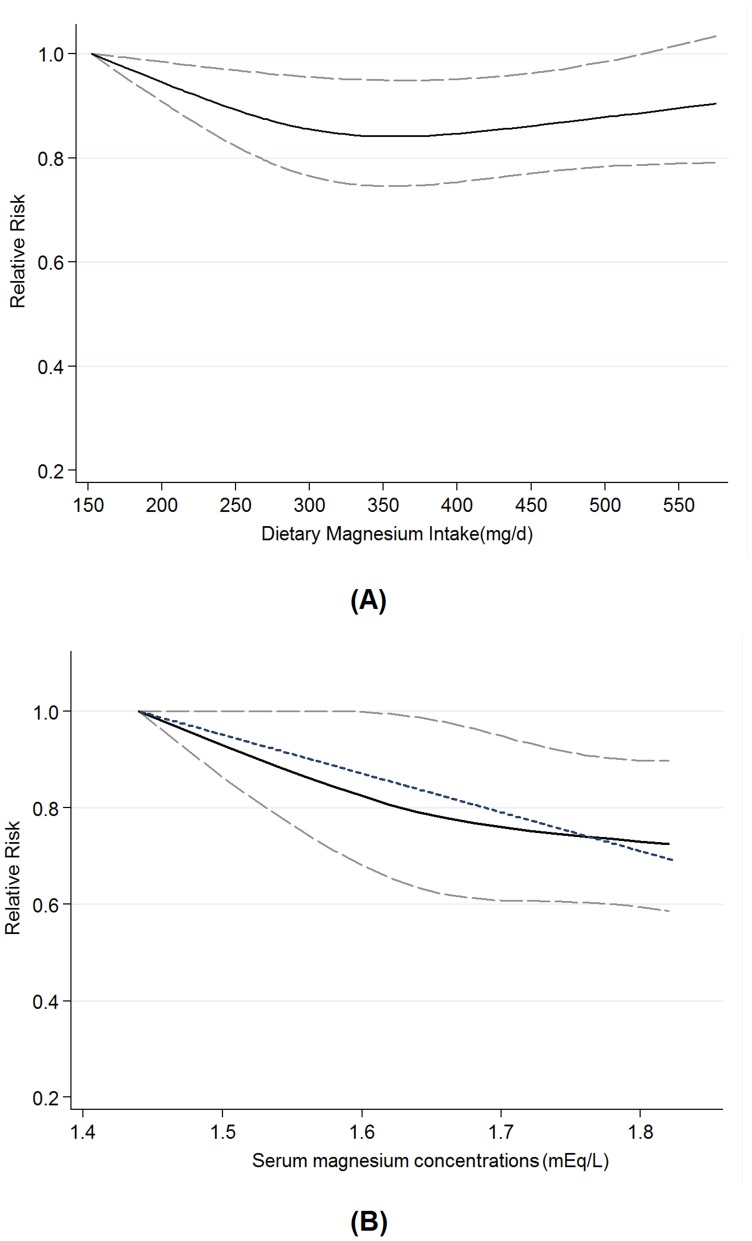
Dose-response relationship between dietary magnesium intake and serum magnesium concentrations and the risk of total CVD events. Relative risk (solid line) with 95% confidence interval (long dashed lines) for the association of dietary magnesium intake and serum magnesium concentrations with risk of total CVD events in a restricted cubic spline random-effects meta-analysis. The short dashed line represents the simpler linear model. The lowest values of 152 mg/d of dietary magnesium and 1.44 mEq/L of serum magnesium were used to re-estimate all relative risks.

We assessed the dose-response relationship between dietary magnesium intake and the risk of total CVD events. Eleven primary studies [18], [20], [22]–[28], [35], [36] were included in this dose-response analysis. The summary relative risk per 100 mg/d was 0.91 (0.86 to 0.96), with moderate evidence of heterogeneity among studies (P* = *0.083, I^2^ = 38.7%). There was evidence of a nonlinear association between dietary magnesium intake and total CVD events risk, P_nonlinearity_ = 0.024, with the greatest reduction for intake between 150 and 400 mg/d but little evidence of further reduction with higher intake (Figure 2). Sensitivity analysis showed that the pooled estimate of the effect of dietary magnesium intake on risk of total CVD events did not vary substantially with the exclusion of any one study.

#### Serum magnesium concentration

Five cohort studies [19], [25], [30], [31], [33] were included in the dose-response analysis of serum magnesium concentrations and the risk of total CVD events. The summary relative risk of total CVD events for every 0.1 mEq/L increment in serum magnesium concentration was 0.91 (0.85 to 0.97), with moderate heterogeneity among studies (P* = *0.011; I^2^ = 63.7%). We found no evidence of a nonlinear relationship between serum magnesium concentrations and total CVD events risk (P_nonlinearity_ = 0.465). The dose-response relation between serum magnesium concentrations and the total CVD events risk is presented in Figure 2. Sensitivity analysis showed that the pooled estimate of the effect of serum magnesium concentrations on risk of total CVD events did not vary substantially with the exclusion of any one study.

## Discussion

### Principal Findings

The findings from this meta-analysis, based on over 532,979 individuals with over 19,900 cases of CVD events, indicate that dietary magnesium intake and serum magnesium concentrations are inversely associated with the risk of total CVD events. A significant association was found between dietary magnesium and total CVD events risk in the nonlinear model. The greatest risk reduction was observed when dietary magnesium intake increased from 150 mg/d to 400 mg/d. An increase of 0.1 mEq/L in serum magnesium concentrations was associated with a 9% decrease in the risk for total CVD events. Analyses stratified by individual CVD outcomes suggest that adequate dietary magnesium intake reduces the risk of stroke, CHD, and CVD death equally. Because of limited information on the individual CVD outcomes, these results should be interpreted carefully and verified by further studies.

During the past 8 decades, dietary and serum magnesium levels have received increased attention and have been the subject of comprehensive studies in cardiovascular health. Magnesium deficiency is considered an important risk factor for different types of CVD. The prevalence of magnesium deficiency is much higher among patients with CVD than among other patients [58], [59]. However, more than half (nearly 65%) of the United States population consumes less than the daily requirement of magnesium from foods [13]. Current guidelines from the World Health Organization (WHO) and several epidemiological studies have demonstrated that intake of magnesium from drinking water may decrease the risk of several types of CVDs [60]–[62]. Nevertheless, compared to magnesium from dietary sources, the amount of magnesium consumed from drinking water is negligible. This fact has weakened the interest in the inclusion of drinking water in preventive strategies for CVD [15]. Dietary magnesium intake is an important component for the primary prevention of CVD.

Several plausible mechanisms have been proposed for the relationship between magnesium and cardiometabolic benefits, including improvement in endothelial function; induction of direct and indirect vasodilation; improved BP; beneficial effects on arrhythmias, inflammatory reactions, and platelet aggregation; and improvement of insulin homeostasis and lipid metabolism [63]–[65]. Furthermore, experimental and epidemiological studies considered that hypertension may serve as an effect modifier of the magnesium and CVD association [16], [17]. According to WHO, 62% of all strokes and 49% of CHD events are attributable to high BP [66]. A previous meta-analysis of 12 randomized clinical trials that tested the effects of magnesium supplementation on BP showed that each 10 mmol/day increase in magnesium was associated with a 4.3 mm Hg reduction in systolic BP and a 2.3 mm Hg reduction in diastolic BP [67]. In the present meta-analysis, most included cohort studies were adjusted for baseline BP or hypertension status; only 1 study [18] assessed the impact of hypertension on the association between dietary magnesium intake and CVD risk. This study found more pronounced associations among hypertensive individuals than among non-hypertensive individuals; this finding supports the beneficial effect of magnesium on CVD outcomes.

Larsson et al [68] conducted a systematic review combining 7 original articles and performed a dose-response meta-analysis to assess the relationship between magnesium intake and stroke risk. They determined that an increase of 100 mg a day in magnesium intake is linearly associated with a 9% decrease in the risk for total stroke (0.88 to 0.97). However, all but 1 [36] of 7 individual studies detected non-significant linear trends between magnesium intake and CVD risk. In contrast to previous meta-analyses, which showed a linear association between magnesium intake and stroke risk, we found evidence of a nonlinear inverse association between magnesium intake and total CVD events risk, with the greatest risk reduction occurring when intake was increased from low levels. Our investigation of the shape of the dose-response curve clarifies this association. This is consistent with the finding of a significant inverse association in our highest vs. lowest meta-analysis.

### Study Strengths and Limitations

To the best of our knowledge, this is the first meta-analysis to estimate the effect of dietary magnesium intake and serum magnesium concentrations on the risk of total CVD events and to include categorical, linear, and nonlinear dose-response meta-analyses. The current meta-analysis had some advantages. First, the number of total participants and CVD events were substantial, which significantly increased the statistical power of the analysis. Second, the quantitative assessment was based on prospective studies, which minimizes the possibility that our findings resulted from recall or selection bias. Third, data extraction, data analysis, and quality assessments of the methods were performed by 2 independent investigators; consistency was checked by arbitrators, contributing to the accuracy of data in the meta-analysis. Finally, there were no publication biases in these meta-analyses; therefore, the entire pooled result may be unbiased.

The possible limitations of our meta-analysis must be considered. First, the quality of the included studies varied, with some having limited adjustments for potential confounding factors. Our study is also subject to confounding factors that could be innate in the included cohorts, which is an inherent weakness of all observational studies and meta-analyses. Although most studies had adjusted for age, BMI, BP, and physical activity for CVD events, confounding by known and unknown risk factors cannot be excluded as a potential explanation for the observed findings [6], [69]. Moreover, it is difficult to completely rule out that the possibility that other nutrients or specific effects of magnesium and other potentially beneficial food components co-existing in the same foods were responsible for the observed association.

Second, some degree of misclassification of exposure may have weakened the strength of the association. Because of the self-administered FFQ of dietary magnesium intake, errors are inevitable. In addition, only 1 report updated the information about dietary magnesium assessment during follow-up [22]. Such errors may have been present in other studies that assessed dietary magnesium at baseline only, which could lead to an underestimation of the relative risk estimates.

Third, due to the lack of data, it is difficult to explain the correlation of dietary magnesium intake with serum magnesium concentrations. In the current systematic review, only 1 study provided information about the inter-relationship between dietary magnesium intake, serum magnesium concentrations, and CVD risk [25]. That study found that individuals in the group with both dietary and serum magnesium greater than the median (serum magnesium = 1.6 mEq/L; dietary magnesium = 241 mg/d) had an approximately 35% lower risk of ischemic stroke than did those in the group with less than median values of both dietary and serum magnesium (relative risk 0.64, 95% confidence interval 0.48 to 0.85), but not in the groups with either dietary or serum magnesium below the median.

Finally, heterogeneity may have been introduced because of methodological differences between studies. After the subgroup and meta-regression analysis, we founded location, sex, and individual CVD outcomes as possible sources of heterogeneity. Although these issues may have reduced the strength of the conclusions drawn in these meta-analyses, visual inspection of forest plots in our meta-analysis suggests that there is considerable consistency in the relative risks across the studies.

### Implications and Directions for Future Research

In the United States, the Recommended Dietary Allowance of magnesium set by the National Institute of Health for men and women aged 31–70 years is 420 and 320 mg/d, respectively. The approximate magnesium content of some foods is as follows: 156 mg in 1 cup cooked spinach, 142 mg in 1 cup cooked soybeans, 122 mg in 2 rectangular biscuits of shredded wheat cereal, and 100 mg in 1 oz roasted peanuts [68], [70]. According to a recent report by WHO, 17.3 million deaths occurred due to CVD in 2008, and this figure will rise to 23.6 million by 2030 [71]. Given that the mortality for total CVD events is 1 in 5, a 15% reduction in the rate of CVD by increasing dietary magnesium intake throughout the population could avert 1–2.5 million deaths from CVD each year [72].

On the basis of this meta-analysis, we believe that future research in the following areas would offer important insights. First, there is a compelling need for the investigation of the inter-relationship between dietary magnesium intake, serum magnesium concentrations, and CVD. To gain information from a mechanistic perspective, an objective assessment of total body magnesium stores and intracellular magnesium concentrations is required [15]. The role of dietary magnesium intake and serum magnesium concentrations should be explained by such an objective assessment. Moreover, several well-designed cohort studies, where both magnesium intake and serum magnesium levels are obtained, with adequate control for confounding factors should be considered. Second, multicenter, double-blinded, placebo-controlled randomized trials should be performed for gaining a better understanding of any causal relationships between magnesium and CVD, especially for sex-specific associations or for individuals at high risk of CVD. Third, it is also important to gain a better understanding of the mechanisms underlying cardiometabolic changes in response to magnesium intake. Investigation of biological and genetic markers may offer additional insights into the role of magnesium in the etiology of CVD.

### Conclusions

In summary, findings from this meta-analysis indicate that dietary magnesium intake and serum magnesium concentrations are inversely associated with the risk of total CVD events. Serum magnesium concentrations ranging from 1.44 mEq/L to 1.8 mEq/L are associated with linear decreases in the risk of total CVD events. There is a nonlinear inverse association between intake of dietary magnesium and total CVD events risk, with the greatest reduction in risk occurring when the intake is increased from 150–400 mg/d. If further interventional randomized controlled trials demonstrate these beneficial effects, new pathways would be opened up for the primary prevention of CVD.

## Supporting Information

Figure S1 **Flowchart of the study selection process.**
(DOC)

Figure S2 **Trim and fill funnel plot for meta-analysis of the association between dietary magnesium intake and serum magnesium concentrations and the risk of total CVD events.**
(DOC)
