# Supplementary material for: Magnesium and the Risk of Cardiovascular Events: A Meta-Analysis of Prospective Cohort Studies
Source: PLoS One. 2013 Mar 8;8(3):e57720. doi: 10.1371/journal.pone.0057720 (PMC3592895; doi:10.1371/journal.pone.0057720)
Supplement: Figure S1 — Flowchart of the study selection process. (DOC) [file pone.0057720.s001.doc]

Used in meta-analysis (n=19)

Retrieved for eligibility (n=35)

Identified from database search (n=2478)

Excluded (n=2443):

Duplicates (n=480)

Did not satisfy criteria (n=1963)

Excluded (n=16):

Cross-sectional (n=13)

Use of odds ratio without CIs (n=3)

Figure S1. Flowchart of the study selection process
