# Supplementary material for: Magnesium and the Risk of Cardiovascular Events: A Meta-Analysis of Prospective Cohort Studies
Source: PLoS One. 2013 Mar 8;8(3):e57720. doi: 10.1371/journal.pone.0057720 (PMC3592895; doi:10.1371/journal.pone.0057720)
Supplement: Figure S2 — Trim and fill funnel plot for meta-analysis of the association between dietary magnesium intake and serum magnesium concentrations and the risk of total CVD events. (DOC) [file pone.0057720.s002.doc]

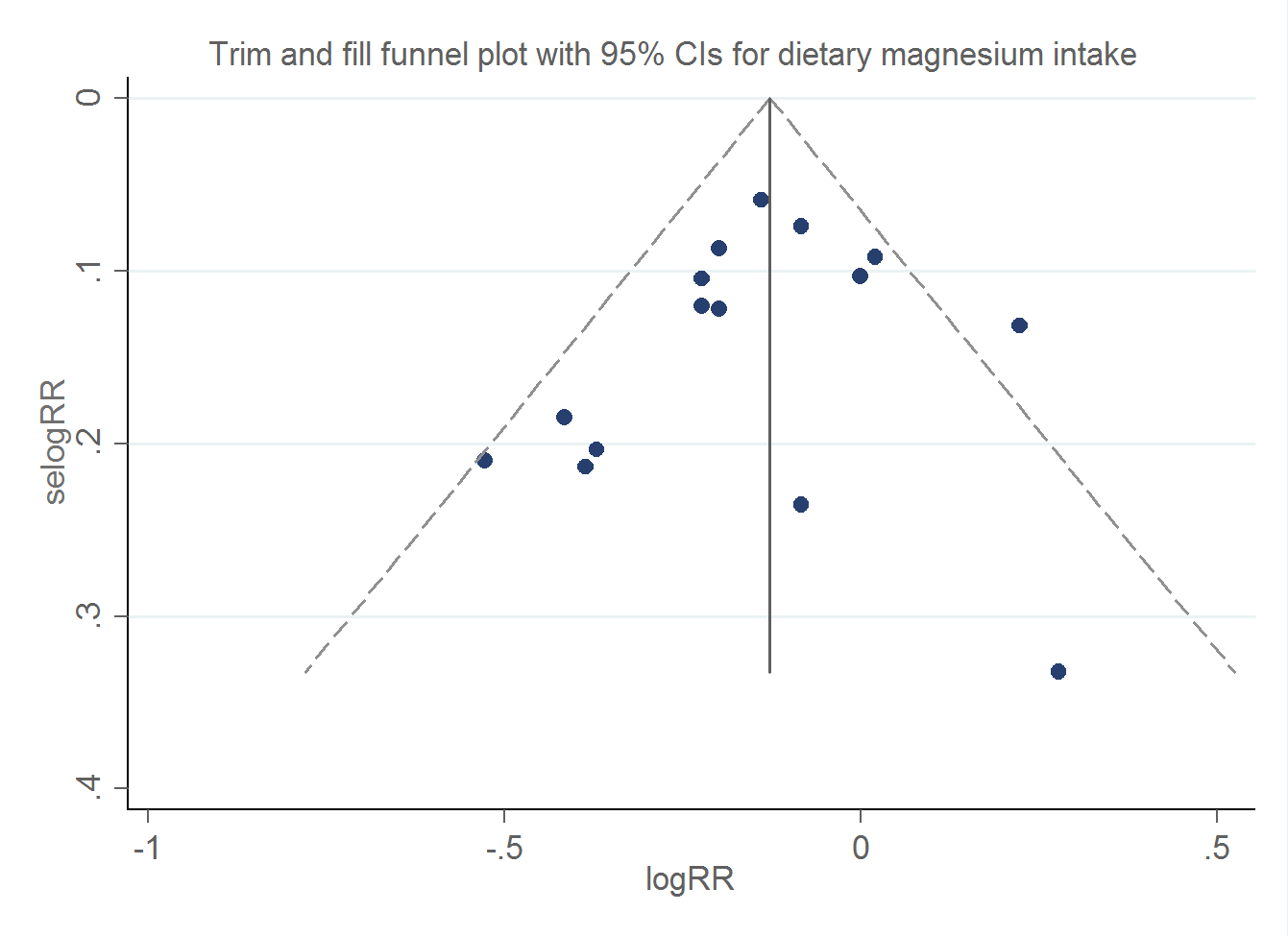


(A)


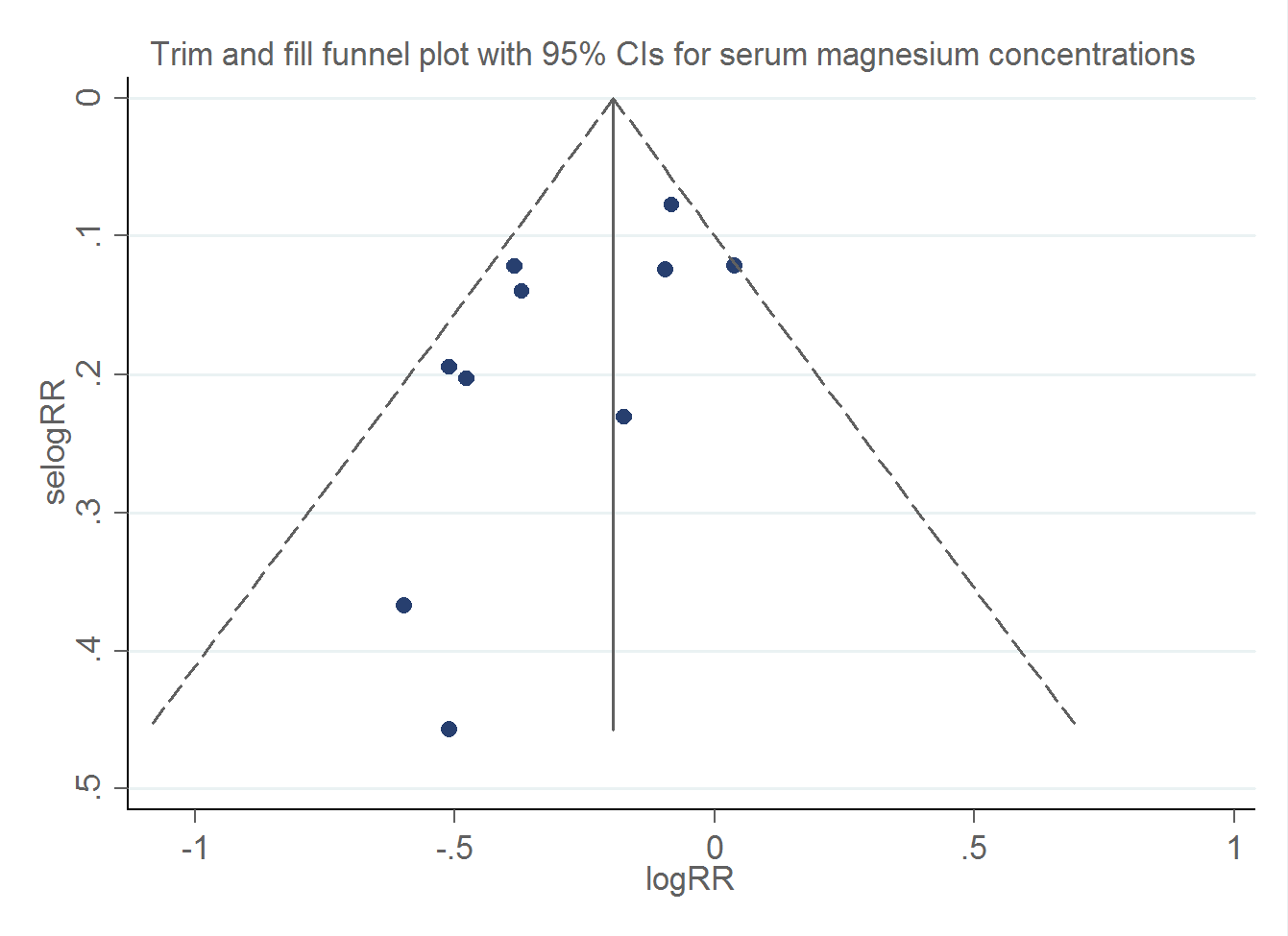


(B)

**Figure S2. Trim and fill funnel plot for meta-analysis of the association between dietary magnesium intake and serum magnesium concentrations and the risk of total CVD events.**

(A) Dietary magnesium intake; (B) Serum magnesium concentrations.

The square represents the hypothetical “missing” studies that might exist and imputed relative risks.
